# Exposed: investigation of oxidation in selenium–tellurium evaporation materials and its effect on optoelectronic devices

**DOI:** 10.1007/s10854-026-17574-5

**Published:** 2026-05-28

**Authors:** Kaitlin Hellier, Thomas D. Yuzvinsky, Evan Walls, Molly McGrath, Shiva Abbaszadeh

**Affiliations:** 1 Radiological Instrumentation Laboratory, Electrical and Computer Engineering Department, University of California, Santa Cruz, Santa Cruz, USA; 2 Electrical and Computer Engineering Department, University of California, Santa Cruz, Santa Cruz, USA; 3 Department of Integrated Engineering, University of San Diego, San Diego, USA

## Abstract

Selenium–tellurium (Se–Te) alloys have attracted sustained research interest due to their combination of photoconductive, thermoelectric, and phase-change properties, which make them promising candidates for a wide range of optoelectronic and energy-conversion applications. However, the long-term stability of Se–Te alloys, especially in bulk, has received little attention. In this work, degradation of Se–Te evaporation materials and its effects on thin-film and device behavior were investigated. Comparative analyses were conducted between Se–Te evaporation pellets stored under ambient conditions for four years and freshly prepared pellets preserved under inert atmosphere. Scanning electron microscopy (SEM) revealed that prolonged atmospheric exposure led to the formation of surface protrusions and crystalline features. Energy-dispersive X-ray spectroscopy and X-ray diffraction indicated surface crystallization and the formation of oxygen-containing phases, consistent with amorphous oxides and trigonal Se. Accelerated environmental aging of new pellets under elevated humidity and temperature replicated similar surface defects, confirming crystallization and oxidation as the dominant degradation pathway. X-ray photoelectron spectroscopy revealed $\hbox {TeO}_x$ as the dominant form of oxide formation, further indicating the formation of amorphous oxides at the surface of the pellets. Devices fabricated from degraded material exhibited increased resistivity, dark-current instability, and suppressed external quantum efficiency, attributable to oxide-induced defects. These results highlight the critical influence of oxidation on Se–Te evaporation material integrity and underscore the necessity of controlled storage and processing conditions for reliable Se–Te-based photodetectors and related electronic applications.

**Supplementary Information:**

The online version contains supplementary material available at 10.1007/s10854-026-17574-5.

## Introduction

Selenium–tellurium (Se–Te) alloys have re-emerged in research interest in the last decade, with a rise in applications in photovoltaics, detectors, memory and switching devices, transistors, and in biomedical applications [1–5]. The compound has been thoroughly studied over the past 75 years for its optical, electronic, and physical properties [6–12].

Alloying Se with Te is known to lower the bandgap of the material, extending photoconduction into the near-infrared, while increasing conductivity [1, 9, 10, 13–18]. Bulk films with Te concentrations greater than 30% typically form a crystalline structure; however, recent studies in thin devices have achieved amorphous behavior with Te content at 40% [19–21]. In amorphous films, the addition of low levels of Te has been shown to improve amorphous structural stability and increase the temperature of crystallization, improving prospects for applications in optoelectronic devices [22–24]. However, these alloys suffer from reduced transport properties, with an increase in defect states induced by Te bonds [25–28]. Even so, Se–Te alloys have been explored with success as broadband sensors, solar cells, and high-gain avalanche rushing photodetectors (HARP), with the latter exhibiting high levels of impact ionization [29].

Largely missing from these studies is discussion of material decomposition and structural stability over time. Amorphous selenium (a-Se) is commonly stabilized through doping with arsenic (As) and chlorine (Cl) to prevent crystallization; however, the long-term efficacy of such stabilization and the conditions for which it holds have had limited study [30–34]. Though it has been noted that alloying a-Se with Te generates minor improvements in stability, especially when compared to As, and many studies observe the effects of photocrystallization in Se–Te, the environmental stability of bulk and thin-film Se–Te systems are few [22, 35, 36].

**Fig. 1:**
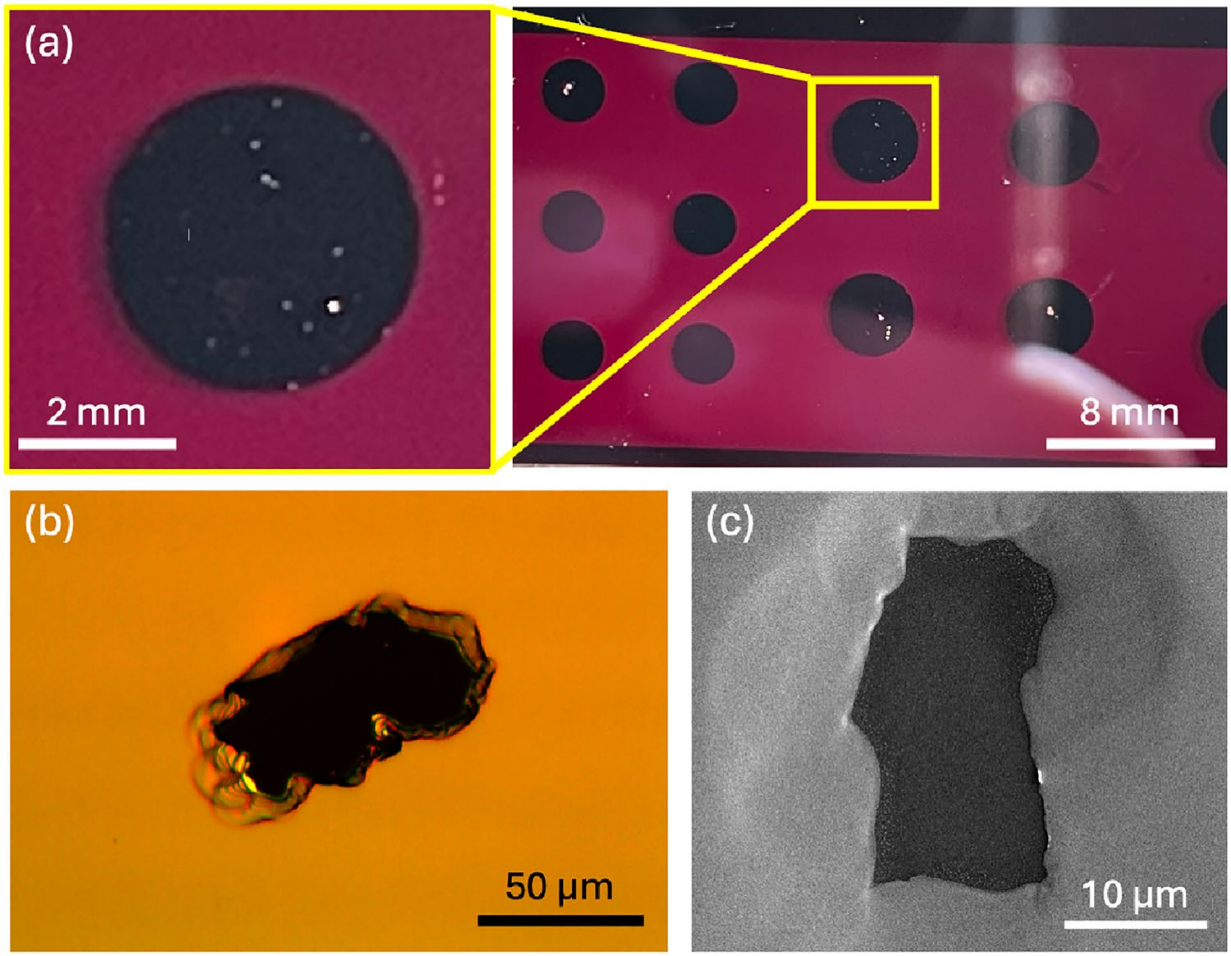
**a** Photo of an Se–Te device, lit from behind, showing voids in the film. The yellow box highlights one device where holes are especially visible. **b** A microscopic image of a film rupture. Ripples at the edges indicate variable material height.** c** An SEM image of another rupture in the surface of a Se–Te film, displaying lifted edges around the rupture

**Fig. 2:**
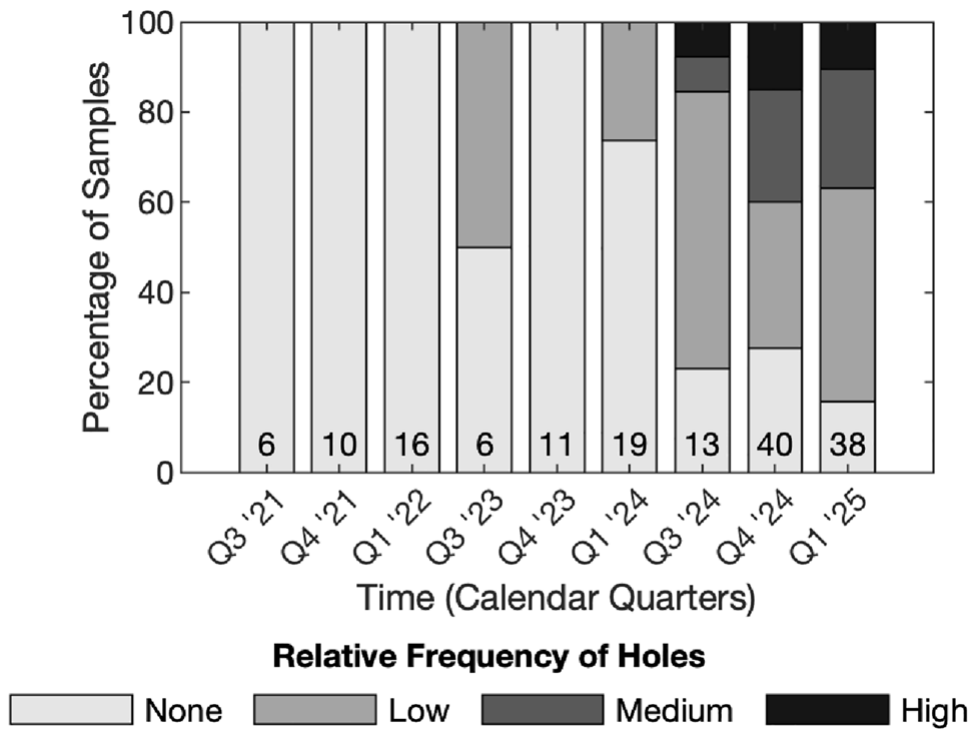
Qualitative histogram of observed defect frequency in Se–Te films fabricated between 2021 and 2024, showing an increase in ruptures over time. The number at the base of each histogram bar represents the total number of samples fabricated in that period. The frequency of defects was determined as low (approximately 1–10 across sample), medium (approximately 10-50 across sample), or high (over 50 across sample)

Se–Te devices fabricated in our lab using pre-alloyed evaporation pellets initially exhibited performance consistent with reported values for similar compositions [37, 38]. However, over time, a progressive degradation in device behavior was observed, including increased failure rates, short-circuiting upon bias application, dark-current instabilities above  20 V/µm, and abrupt current surges at higher fields (40–60 V/µm), indicative of field-induced breakdown rather than photogeneration. Post-fabrication inspection revealed surface perforations and voids; Fig. 1a displays the underside of a representative device, where numerous perforations are visible through the top contact. Subsequent optical and scanning electron microscopy (SEM) (Figs. 1b,c) revealed material gaps, raised edges, and localized roughening consistent with rupture events and possible gas evolution. Visual analysis, including of untested devices and films, demonstrated a steady increase in defect density over multiple years, with no correlation to fabrication parameters (e.g., user, substrate temperature/type, environment, or film thickness). Figure 2 demonstrates the timeline of defect appearances. These defects were exclusively observed in Te-containing samples, and process modifications—including substrate cleaning, deposition rate variation, and extended pre-deposition evaporation—did not mitigate their formation. The only consistent variables were Te incorporation and time-dependent exposure, suggesting environmental degradation of the source material as the dominant mechanism.

New evaporation pellets were ordered, packed under inert gas and in smaller units, to assess the condition of the materials. To determine the role of the storage condition and how the original Se–Te materials were impacted over time, a comparative study of the old and new Se–Te evaporation pellets was performed. This work examines the changes observed in the pellets and the impact they had on the films and devices fabricated from the materials.

## Methods

### Materials and fabrication

Pre-alloyed Se–Te pellets were purchased from William-Rowlands with a 70 wt. % Se-to-30 wt. % Te ratio; pellets were fabricated by the manufacturer by establishing a high-purity selenium–tellurium melt, granulating it through a sieve into deionized cold water, then vacuum drying the resultant pellets at low temperature to prevent crystallization. All work were performed in an argon glovebox. The initial batch (Batch A, delivered May 2021) was supplied in a large air-packed plastic container and stored under ambient conditions in the dark when not in use. The second batch (Batch B, May 2025) was packaged in 100 g argon-sealed bags and retained in its original airtight packaging until use. Upon opening, pellets were transferred to a crucible in the thermal evaporator for immediate use unless used for immediate testing. The residual melt-material post-deposition was left under vacuum conditions except during chamber preparation.

Thin films were prepared by evaporation of the Se–Te pellets in a dedicated Se thermal evaporator onto ITO-coated glass substrates (Delta Technologies, Ltd; ITO: 70–100 $\Omega$/sq, 15–30 nm thick). Substrates were rotated at 40 rpm during deposition and either maintained at room temperature or heated to 60 $^\circ$C to control film morphology. Deposition rates were maintained between 90 and 105 Å/s at base pressures below $1.5 \times 10^-6$ Torr, yielding films of approximately 3 µm thickness.

Devices were fabricated on ITO/glass substrates with and without a 30 nm silicon dioxide ($\hbox {SiO}_2$) hole blocking layer, deposited by electron beam evaporation. Gold top contacts of 100 nm were deposited by electron beam evaporation, utilizing a shadow mask to pattern 3- or 4-mm diameter devices.

#### Characterization

Optical microscopy was conducted using Nikon objectives (5x and 20x) with digital images captured via a Moticam 3.0 camera. Low-resolution photos were captured by a phone camera (iPhone X 10) under white LED back-illumination.

Surface and cross-sectional images of pellets and films were taken using a Thermo Scientific Scios 2 DualBeam SEM operated at 10–20 kV and 25–80 pA. Pellets were cleaved using a razor blade to initiate fracture, allowing natural propagation through the bulk to minimize mechanical damage. Images were acquired in regions unaffected by direct blade contact, avoiding visible striations or mechanical deformation artifacts. Elemental analysis was performed using an Oxford Instruments AZtecLive Ultim Max 100 energy-dispersive spectroscopy (EDS) detector with the electron beam operating at 10 kV and 0.80 mA beam current. Transition energy levels were reported from those provided by the AZtecLive analysis procedure and verified against database values [39, 40].

Accelerated aging of Batch B was conducted under high-humidity and elevated-temperature conditions. Pellets were mounted on SEM stubs using carbon adhesive tape, imaged, and then placed in a polystyrene sample box. Deionized (DI) water was introduced to maintain humidity without direct liquid contact with the samples. The box was closed and kept on a hot plate at 50 $^\circ$C for three weeks, besides the time taken for testing, to accelerate environmental interaction. The stub was removed and pellets imaged by SEM in the same location each week after the first imaging was performed, and DI water was added as necessary to maintain a humid environment. During the third week, no additional water was introduced to evaluate the effect of reduced humidity on surface modification.

X-ray diffraction (XRD) was performed on Batch A, Batch B, and Batch B (aged) pellets to compare composition. Pellets were ground into coarse grains and mounted on a rough quartz substrate designed for powder diffraction. Measurements were taken by a Rigaku SmartLab X-ray Diffractometer over a 2$\theta$ range of ${10}^\circ$ to ${70}^\circ$, scan rate ${1}^\circ$/min, step size of ${0.02}^\circ$, and source parameters of 45 kV and 200 mA.

X-ray photoelectron spectroscopy (XPS) was performed by Eurofins EAG Laboratories on Batch A, Batch B, and Batch B (aged) pellets. Characterization was carried out on a Thermo K-Alpha Plus XPS spectrometer with a monochromated AlK$\alpha$ X-ray source. Survey scans were collected over a 400 µspot size from 1350 to 0 eV binding energy at a pass energy of 200 eV, with 1 eV step size and a total of 4 scans carried out with a scan rate of 20 eV per second. High-resolution scans were collected with a pass energy of 50 eV and step size of 0.1 eV for the Se 3d, Te 3d5, O 1 s, and C 1 s + Se LMM spectral ranges. A charge correction was implemented using the Se 3d5 energy of 55.1 eV. The data were analyzed using a non-linear least squares curve fitting to the high-resolution spectra.

Devices fabricated from Batch A and Batch B were characterized for dark and photocurrent at a range of applied fields utilizing a Keithley 6487 picoammeter. The external quantum efficiency (EQE), $\eta$, was calculated from

1 $$\eta = \frac{{(I_{P} - I_{D} )/e}}{{P_{\lambda } *{\lambda } /hc }}$$

where $I_P$ is the average photocurrent, $I_D$ is the average dark current, *e* is the elementary charge, $P_\lambda$ is the power incident on the device, $E_\lambda$ is the wavelength of light, *h* is Planck's constant, and *c* is the speed of light. Devices were illuminated with monochromatic light ($\lambda$ = 533 nm) at intensities of 0.4–0.7 µ$\hbox {W/cm}^2$ for 1 s; photocurrent was averaged over this period. Each measurement cycle consisted of a dark-settling period after bias ramping, followed by illumination and a subsequent dark recovery to baseline current. Bias was increased from one voltage to the next, with dark-settling and illumination steps repeated at each level to ensure steady-state response before proceeding to the next field. A minimum of three devices were characterized to ensure consistency in performance. Film thicknesses were found by stylus profilometry (Dektak 3).

## Results and discussion

**Fig. 3:**
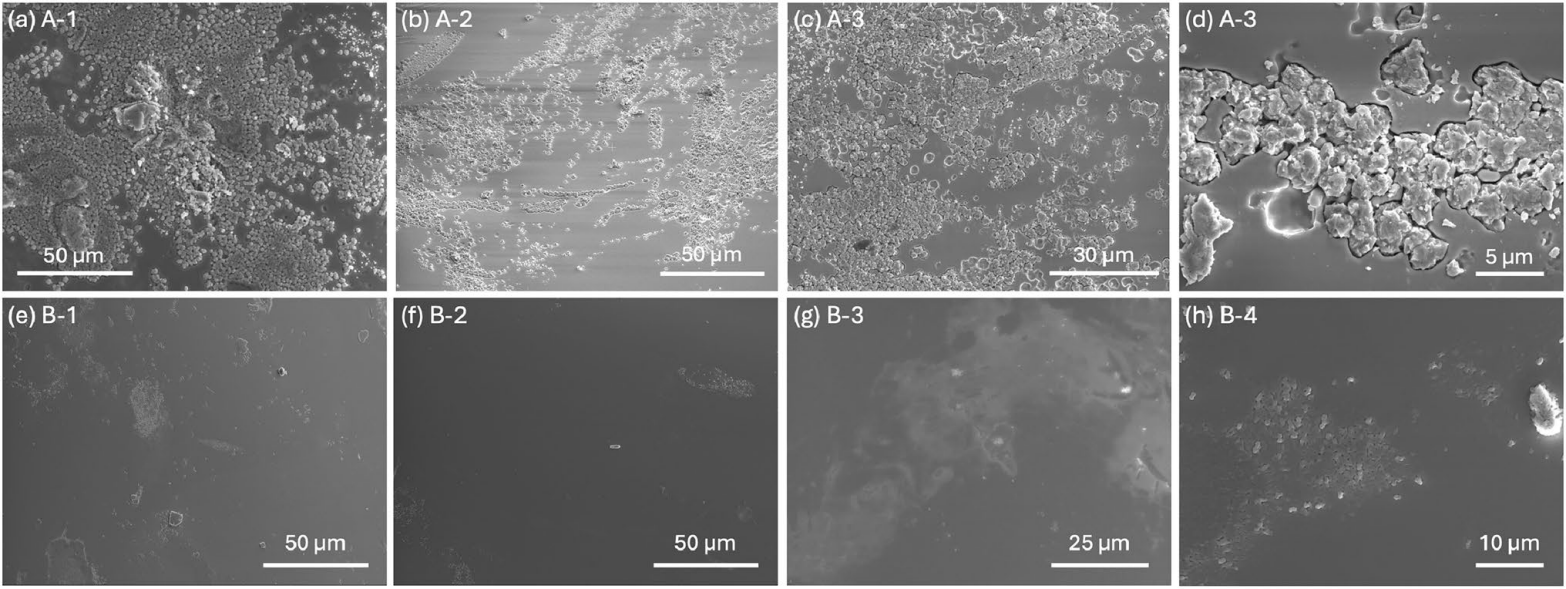
SEM imaging of pellets from **a**–**c** Batch A and **d** a closer look at the surface of one of the Batch A pellets, showing rough structures in and protruding from the surface. **e**–**g** SEM images of Batch B pellets

An image of Batch A and Batch B pellets can be seen in Fig. S1. Pellets are roughly toroidal, about 1–4 mm across, with variations in size and appearance. Se–Te pellets with a relatively flat surface were selected for SEM imaging. Figure 3a–c shows images from three different pellets of Batch A. While some areas are smooth, protrusions spread across the surface. Upon closer inspection, as seen in Fig. 3h, these exhibit a more structured morphology, possibly indicating the onset of crystal formation at the surface of the material. This behavior was noted across all of the Batch A samples, including additional samples shown in Fig. S2. Figure 3e–h shows images of Batch B across four different pellets. The surfaces are predominantly smooth, with occasional small raised areas, scratches, or semi-fused patches at the surface, likely where the original Se and Te elemental material did not fully combine. These features lack the faceting or roughness seen in Batch A, and still appear largely uniform and featureless.

**Fig. 4:**
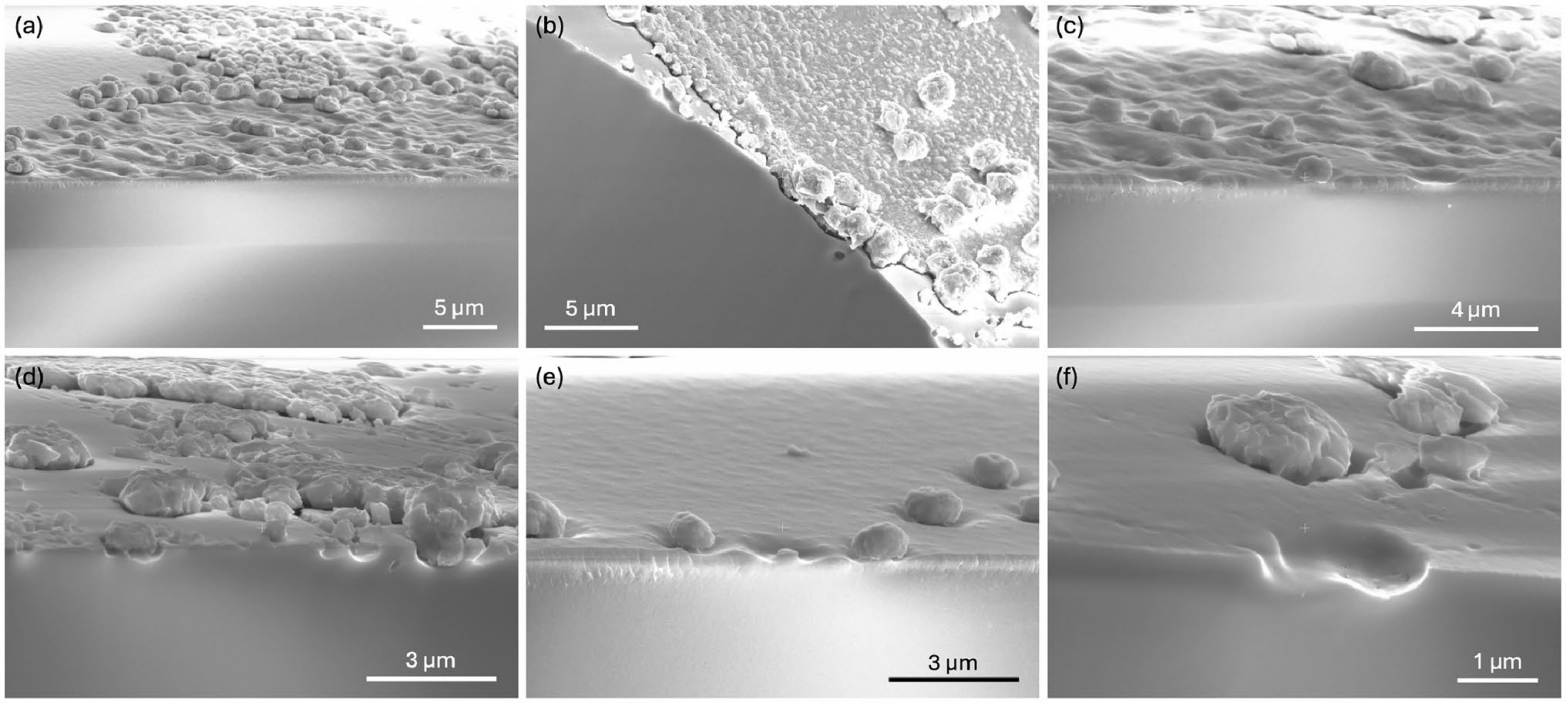
Cross-sectional SEM of Batch A pellets cleaved in half. All images show a largely distorted surface compared to the inner portion of the pellet, with protrusions that resemble florets emerging, frequently in clusters

To better observe the structure and behavior of these protrusions, multiple Batch A pellets were cleaved in two, allowing for cross-sectional SEM of the surface formations. Figure 4a–f shows images captured from two cleaved pellets. In all cases, distortions to the structure, including protrusions and rough material, occur only at the surface. The cleaved edges in Figs. 4d–f show that the protrusions occur like a floret; there is a stub from which the rough, rounded material forms, becoming segregated from the main body of the pellet. Figures 4e and 4f show where the cleaving removed the top of the floret, leaving a stub (4e) and a cavity (4f) behind. This implies that the contamination at the surface extruded elements from the pellet, formulating new structures, and possibly new compounds.

**Fig. 5:**
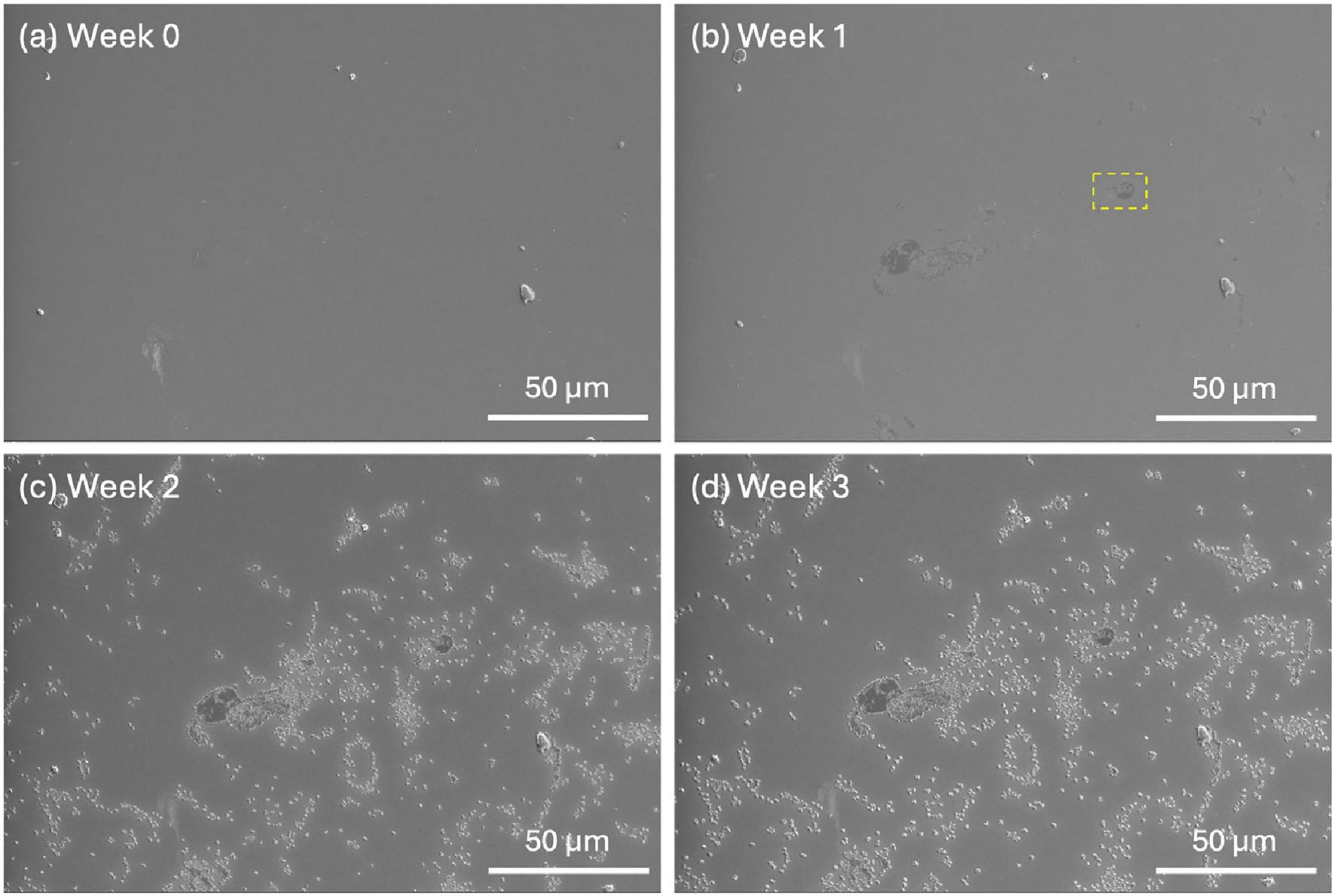
SEM images of a Batch B pellet taken in roughly the same position** a** prior to exposure, **b** after one week of aging,** c** after two weeks of aging, and** d** after three weeks of aging. The yellow box in** b** highlights the area of focus in Fig. 6. Image contrast and brightness were adjusted to match portions of the smooth area for better comparison

To test if the presence of atmospheric water assisted in the development of the surface crystals in Batch A, the most common contaminant to which it had exposure, accelerated degradation was performed on Batch B using a high-humidity, high-temperature environment. After SEM imaging the fresh material, the pellets were aged and measured in the same location once a week for the next two weeks; the third week of aging remained on the hot plate, without additional DI water added. Figure 5 shows the evolution with time for one pellet; the other four pellets can be seen in Fig. S3. After one week in the high-humidity environment, blemishes appear on the surface of the pellet, appearing as discolorations in the images. The surface appears slightly rougher around these areas and near other protruding masses. By the second week, there are a large number of protrusions scattered across the surface, most commonly following the edges of a blemish or existing protrusion, or amassing in a cluster, occasionally with some directionality. After the third week of exposure in reduced humidity, the surface does not appear to have gained more defects, though they have become slightly more defined.

**Fig. 6:**
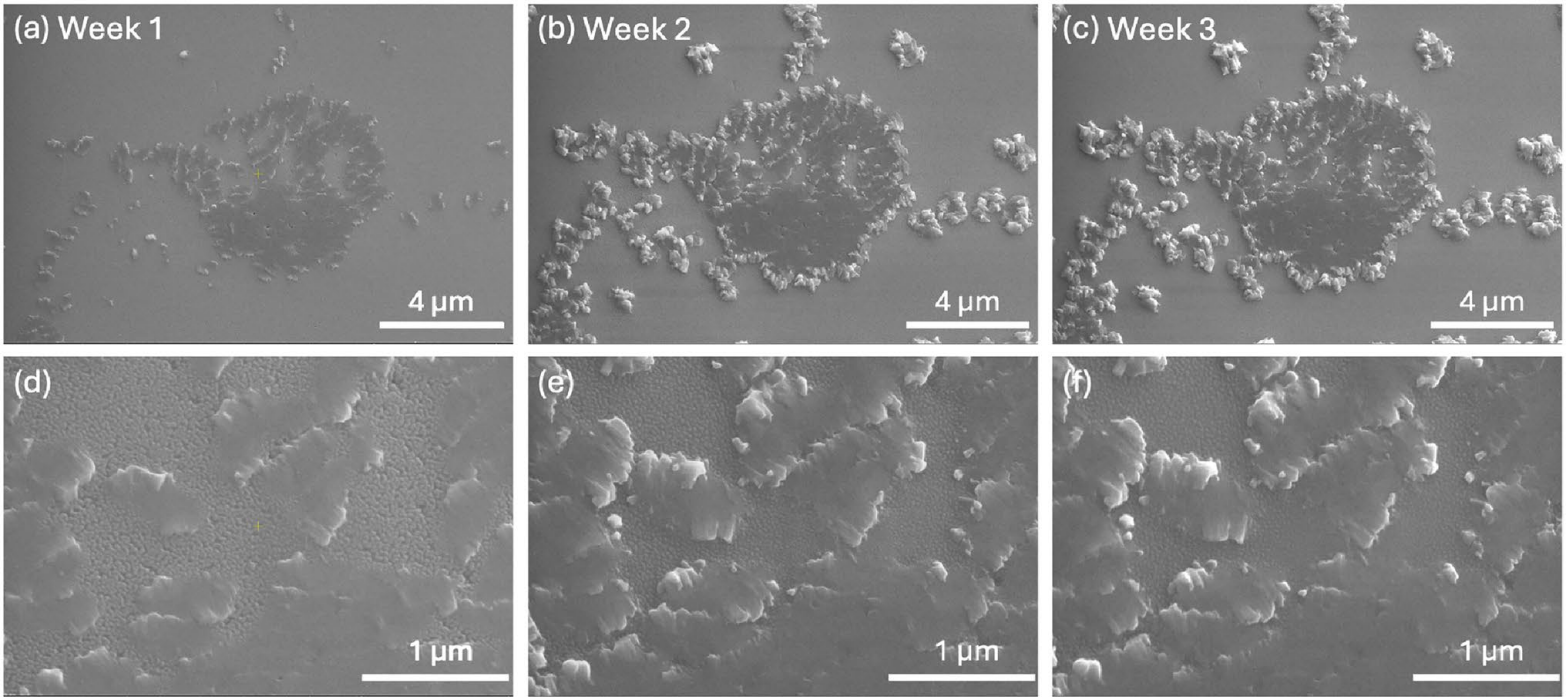
Development of the blemish highlighted in Fig. 5b after** a** one week of aging,** b** two weeks of aging, and** c** a third week of aging in reduced humidity.** d**–**f** A closer look at the center area of the image above over the same time progression

A closer look at one of the blemishes that appeared on the surface where none existed prior to accelerated degradation (highlighted in Fig. 5b with a dashed yellow box) can be seen in Fig. 6. The top images correspond to the successive weeks of aging; the bottom images focus on the region at the center of the images above. From one week to two weeks of aging (Figs. 6a and 6b), we see a high amount of development, as the protrusions rise from the surface and become much sharper along the edges. Going into the third week, we see not much changed, aside from slightly higher definition along the edges of the protrusions.

Comparing these images to those of Batch A (Figs. 3e–g and 4), we can see that the defects of the accelerated aging are less numerous, smaller, and more jagged. The Batch B (aged) defects also sit atop the surface, as opposed to being embedded as they are in Batch A, and lack the rounder, floret-like characteristics. This may be due to Batch B (aged) being in the early stages of degradation, or the accelerated interactions that were induced compared to the slow degradation that occurred in Batch A.

**Fig. 7:**
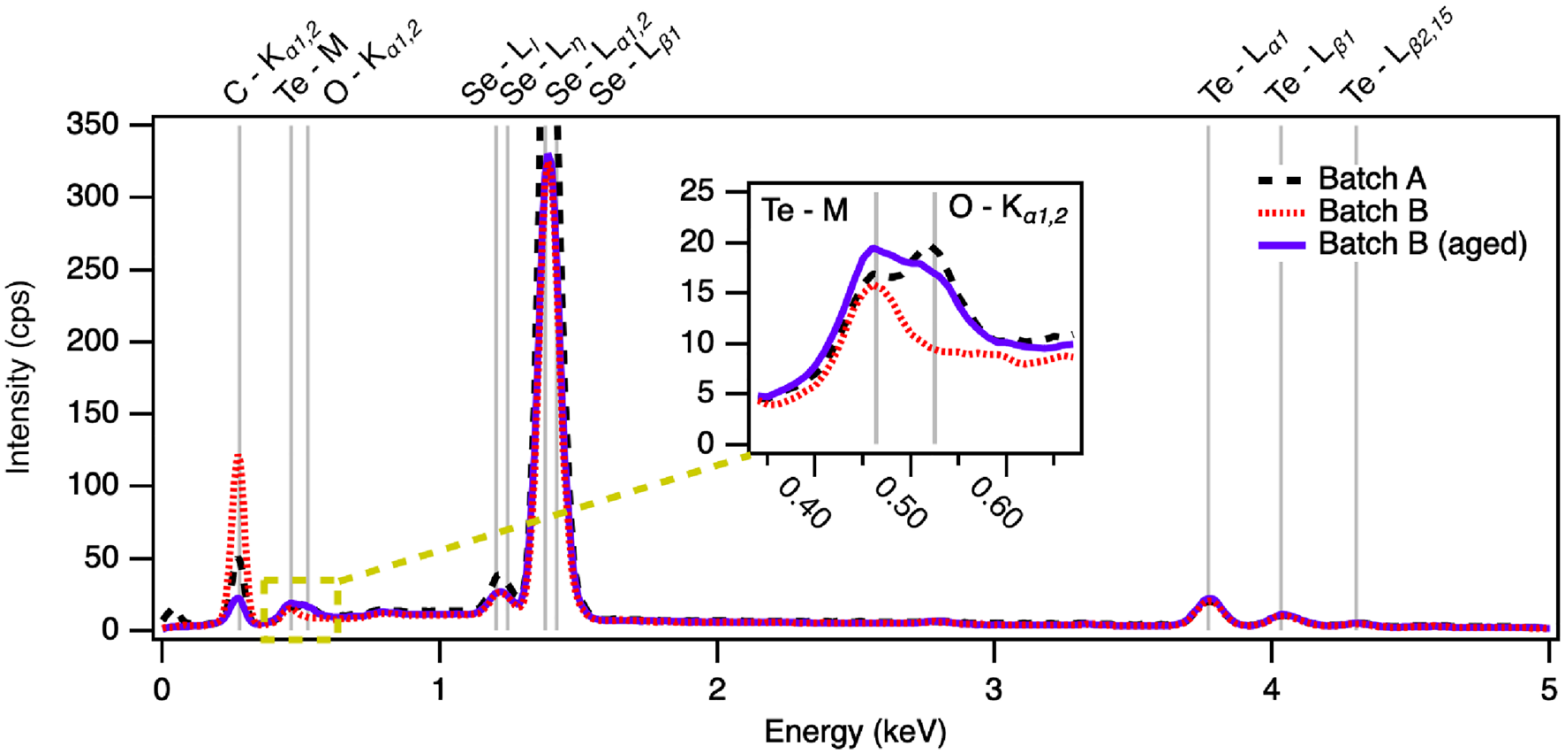
Typical EDS spectra of Batch A, Batch B, and Batch B (aged). Gray vertical lines mark transitions assigned to each peak. The inset highlights the region around the Te–M and O–$\hbox {K}_{\alpha 1,2}$ transitions

Elemental compositions for Batch A, Batch B, and Batch B (aged) pellets were determined by EDS; these values may be found in Table S1 in the SI. Figure 7 shows typical spectra taken from each, with aged composition taken after the study was complete. Peak identifications show clear spectral lines from Se, Te, and carbon (C), the latter likely occurring from contamination during mounting to the carbon adhesive tape. All three pellets show a subtle peak from the Te–M transitions, highlighted in the inset. Batch A and Batch B (aged) pellets also show a shoulder encompassing 0.525 keV, the K$_{\alpha 1,2}$ transition for oxygen, while the spectra for Batch B do not. The composition places this value around 0.6−2.0 wt. % depending on the location, area scanned, and definition of the O shoulder in fitting. A definitive value for any sample was difficult, given the limited number of peaks distinguishable in EDS for oxygen and the inhomogeneous oxidation of the pellets; however, it is clear that there is oxygen present in the Batch B (aged), where there is not in the un-aged Batch B.

**Fig. 8:**
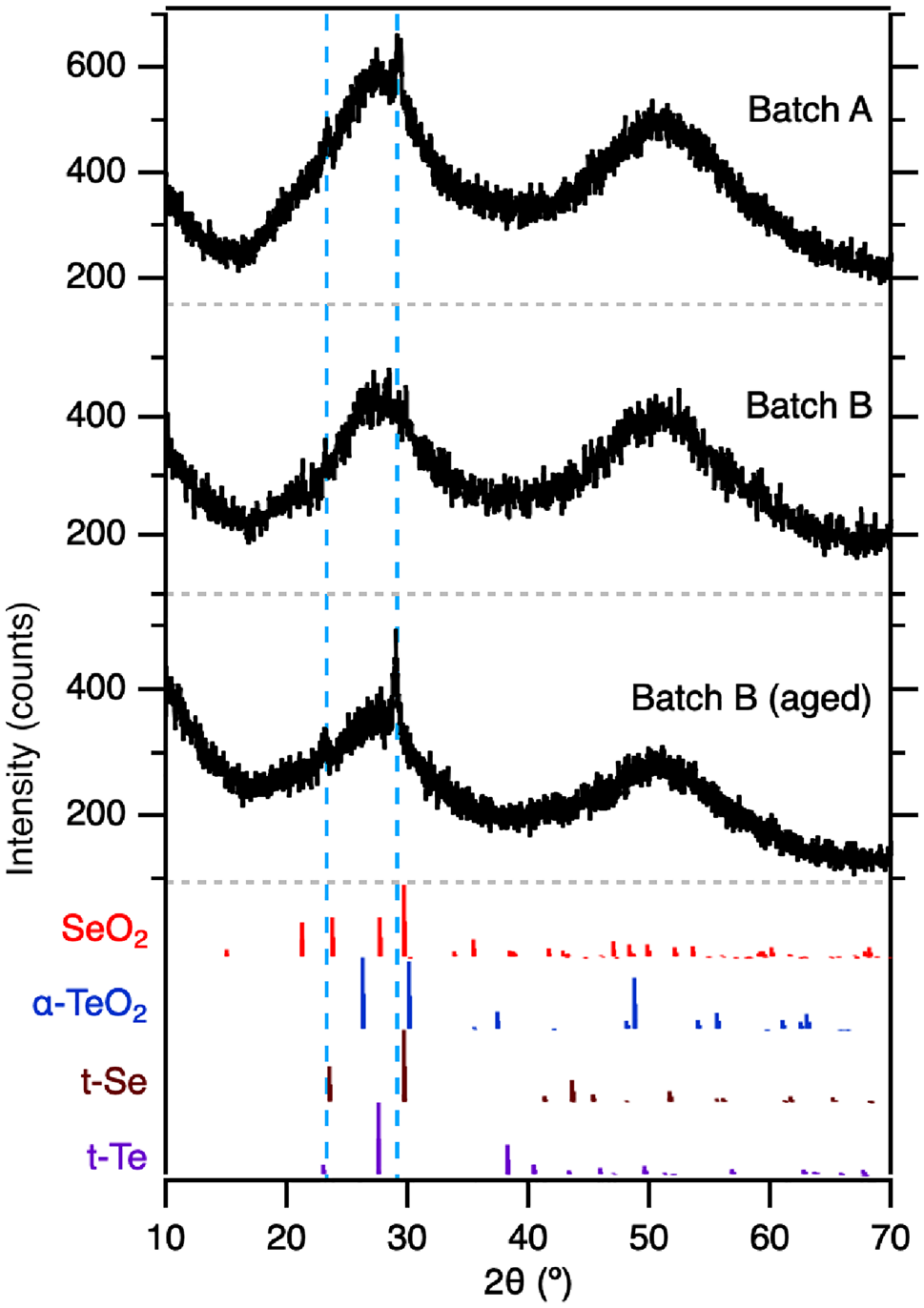
X-ray diffraction spectra for the crushed pellets. Dashed blue lines highlight the peaks formed in the Batch A and Batch B (aged) samples. Reflections for t-Se (ICSD-40018), t-Te (ICSD-161690), $\hbox {TeO}_2$ (ICSD-56004), and $\hbox {SiO}_2$ (ICSD-72366) are shown in the bottom quarter of the plot

Powder XRD of the crushed pellets for Batch A, Batch B, and Batch B (aged) can be seen in Fig. 8. Batch B exhibits an amorphous structure with broad peaks around ${27}^\circ$ and ${51}^\circ$, similar to that seen in a-Se, with no discernible sharp peaks. Batch A shows two small peaks among the amorphous behavior, at $\hbox {23.3}^\circ$ and $\hbox {29.1}^\circ$. Batch B (aged) also exhibits these peaks, with the ${29.1}^\circ$ achieving greater differentiation and a sharper appearance. In both cases, we can expect any peaks apparent to be small; given that defects were only at the surface, the amount of crystalline material will be very small compared to the rest of the amorphous pellet.

Crystalline compounds involving Se, Te, O, and H were explored for peak matching in the ICSD database; reflections for the most common structures of crystalline t-Se (ICSD-40018), t-Te (ICSD-161690), $\hbox {TeO}_2$ (ICSD-56004), and $\hbox {SiO}_2$ (ICSD-72366) are shown at the bottom of the plot in Fig. 8 [41–45]. No other feasible recorded compounds or structures aligned with the peaks exhibited. The peaks show good alignment with t-Se; in the case of $\hbox {SeO}_2$, the (201) reflections around $\hbox {27.7}^\circ$ have similar intensity around to the (210) reflections around ${23.8}^\circ$, but are not seen in the scans. Another possibility, though unlikely: $\hbox {SeO}_2$ nanoparticles have been shown to exhibit peaks similar to t-Se, with slight peak 2$\theta$ angle fluctuations dependent on synthesis [46, 47]. Oxygen may also be present as an amorphous form of $\hbox {SeO}_2$ or $\hbox {TeO}_2$, inducing crystallization and extrusion of Se at the surface of the pellets. It is clear, however, that crystalline H$_2$SeO$_3$ or H$_2$SeO$_4$ have not formed and remained within the material.

**Fig. 9:**
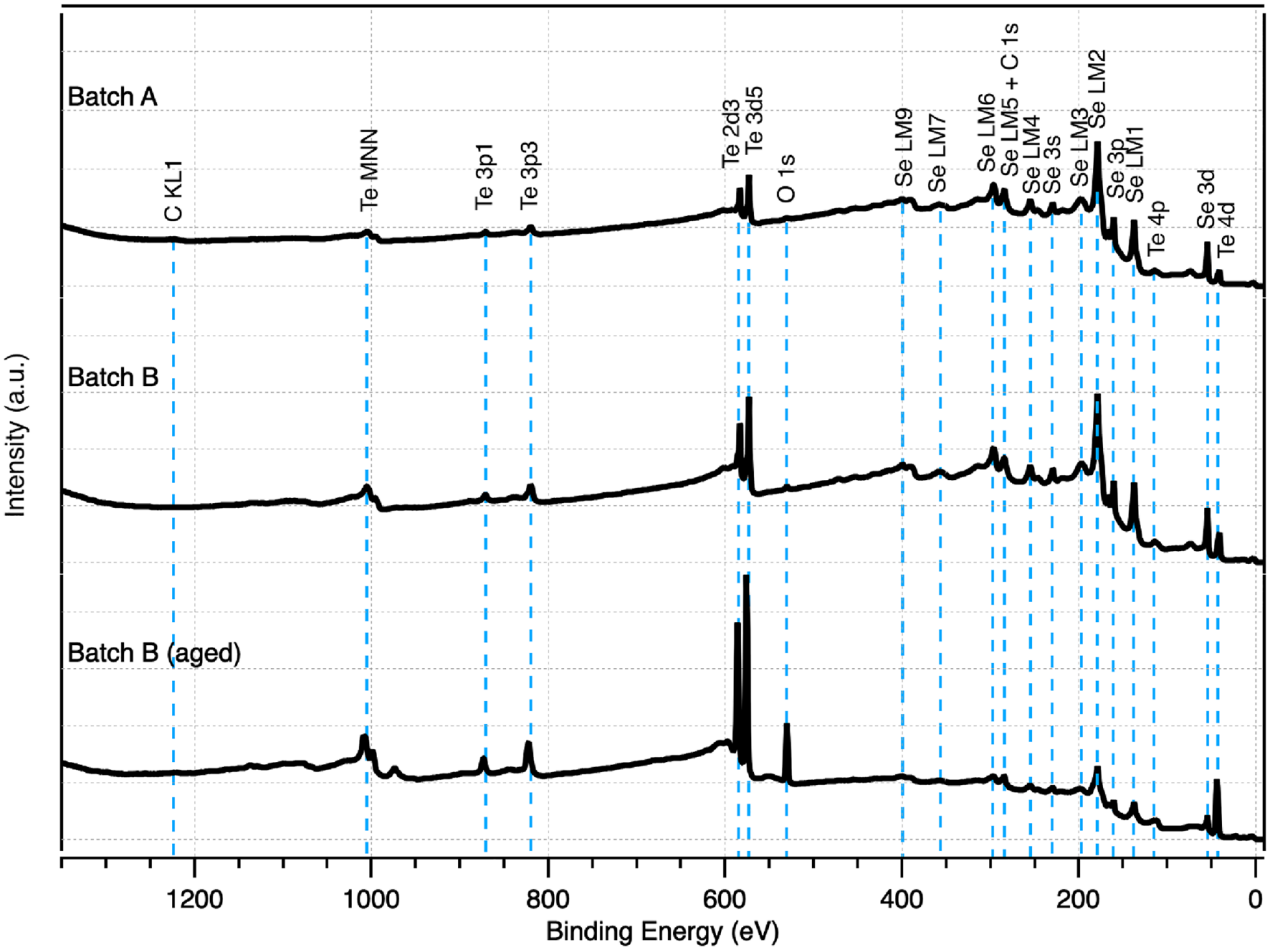
XPS spectra for Batch A, Batch B, and Batch B (aged), with noted peaks marked above Batch A and dashed blue lines guiding the eye to the associated binding energies

Samples from the three batches of pellets were sent for XPS analysis to gain a more accurate concentration of oxygen content and bonding structures at the surface. Characterization only occurs within the top 5–10 nm of the surface, allowing a detailed investigation absent bulk properties. Figure 9 shows the full survey scan of the three pellets; the high-resolution scans of the Se 3d, Te 3d5, O1s, and C1s + Se LMM can be found in Fig. S4 of the SI. Table 1 shows the atomic concentrations of detected elements for each batch. The sample surfaces were primarily composed of Se, Te, $\hbox {SeO}_x$, and $\hbox {TeO}_x$, with oxygen preferentially bonding to Te. Carbon was found at moderate levels on the Batch A sample, with a small amount on Batch C. This is likely due to organic contaminants from air exposure, as Batch B showed no presence of the element. All samples showed the presence of oxygen, indicating that, despite fabrication in a glovebox environment, the pellets are highly susceptible to contamination and even small traces may lead to some level of surface contamination. The lack of additional exposure prevented deeper incursion of oxygen into the Batch B pellets, leaving traces only at the surface and undetectable by EDS due to the high penetration depth. The variation in Se–Te compositions of Batch A and Batch B samples indicates that some level of Se elemental extrusion occurs during the surface oxidation of the Se–Te surface, increasing with time and exposure. The decrease in the Se-to-Te ratio in the Batch B (aged) sample suggests possible Se depletion at the surface, potentially associated with moisture-assisted oxidation and the formation of hydrated selenium oxide species (e.g., $\hbox {H}_2\hbox {SeO}_3$), which may exhibit increased mobility under mildly elevated temperatures [48].

Table 2 shows the ratio of chemical states related to Se/SeO$_x$ and Te/TeO$_x$. Se and Te were predominantly found as $\hbox {Se}^0$ and $\hbox {Te}^0$, with the $\hbox {Te}^0$ found at a slightly higher binding energy than expected. Oxygen did not show presence in any states besides that of metal oxides, and the majority of bonds were with Te atoms across all samples. The lack of visibility in the XRD data of Fig. 8 implies that these oxides are amorphous. While some oxide formation occurs with Se in Batches A and B, it is minimal. Batch B (aged) shows a much higher level of oxidation, with the majority of Te chemical states associated with TeO$_x$, almost fully converting the surface Te atoms to oxides. A higher level of SeO$_x$ bonds are also present, likely due to a much more developed defect space and opportunities for interaction. These results show a clear picture of what oxides are formed in the pellets and how the structure is altered.

**Table 1:** Atomic concentrations of detected elements in each pellet reported from XPS, listed as a percentage of the total concentration

Sample	C	O	Se	Te
Batch A	19.5	4.2	66.5	9.8
Batch B	-	5.6	74.1	20.3
Batch B (aged)	2.6	52.9	19.4	25.2

**Table 2:** Chemical states of each element for each pellet sampled

Sample	Se, in total % Se		Se, in total % Se	
	$\hbox {Se}^0$	SeO$_x$	$\hbox {Te}^0$	TeO$_x$
Batch A	99	1	80	20
Batch B	98	2	83	17
Batch B (aged)	85	15	3	97

To understand the effects of the oxygen contamination in photodetectors, the end goal of this work, devices were fabricated from both Batch A and Batch B. Selenium and tellurium have melting points of 221 $^\circ$C and 449.5 $^\circ$C, respectively; the alloy of the two falls somewhere between [49]. To ensure amorphous and high-quality films, the deposition rate was held around 90 Å/s, requiring temperatures of 290-330 $^\circ$C for evaporation. This range encompassed the temperature of sublimation for SeO$_2$ at 315 $^\circ$C; it does not, however, include TeO$_2$. This resulted in films with a Te concentration of 11–16 wt. %, and a small concentration of O in the Batch A films around 0.1−0.6 wt. %. Examination of the remaining melt-material from Batch A after cooling showed a dull, yellow-tinged coloring across the surface of the Batch A Se–Te, while the Batch B material remained a dull, dark silver, as seen in Fig. S5. This is consistent with the presence of oxides in Batch A, as SeO$_2$ and TeO$_2$ have been reported as white solids, with some yellow coloration possible [50–53]. The resultant films demonstrated uniform thickness across the sample, similar to that established for this system in previous works. [54].

The variation in Te concentration arises from the difference in Se and Te melting temperatures; the Se will evaporate at a faster rate than the Te, leaving the remaining material higher in Te concentration. Before and after deposition of the film, a large amount of material is evaporated during the heating and cooling of the crucible. While we do not see high levels of variation in the concentration through the thickness of the film, we do see an increase in Te concentration from subsequent depositions that have not had additional material added. The high variance in O content in the Batch A films may arise from additional oxidation nucleation points at the surface, influenced by the defects formed. Additionally, the analysis software used may have struggled to quantify the level of oxygen content as it approaches the limitations of system, providing different values based on fit.

**Fig. 10:**
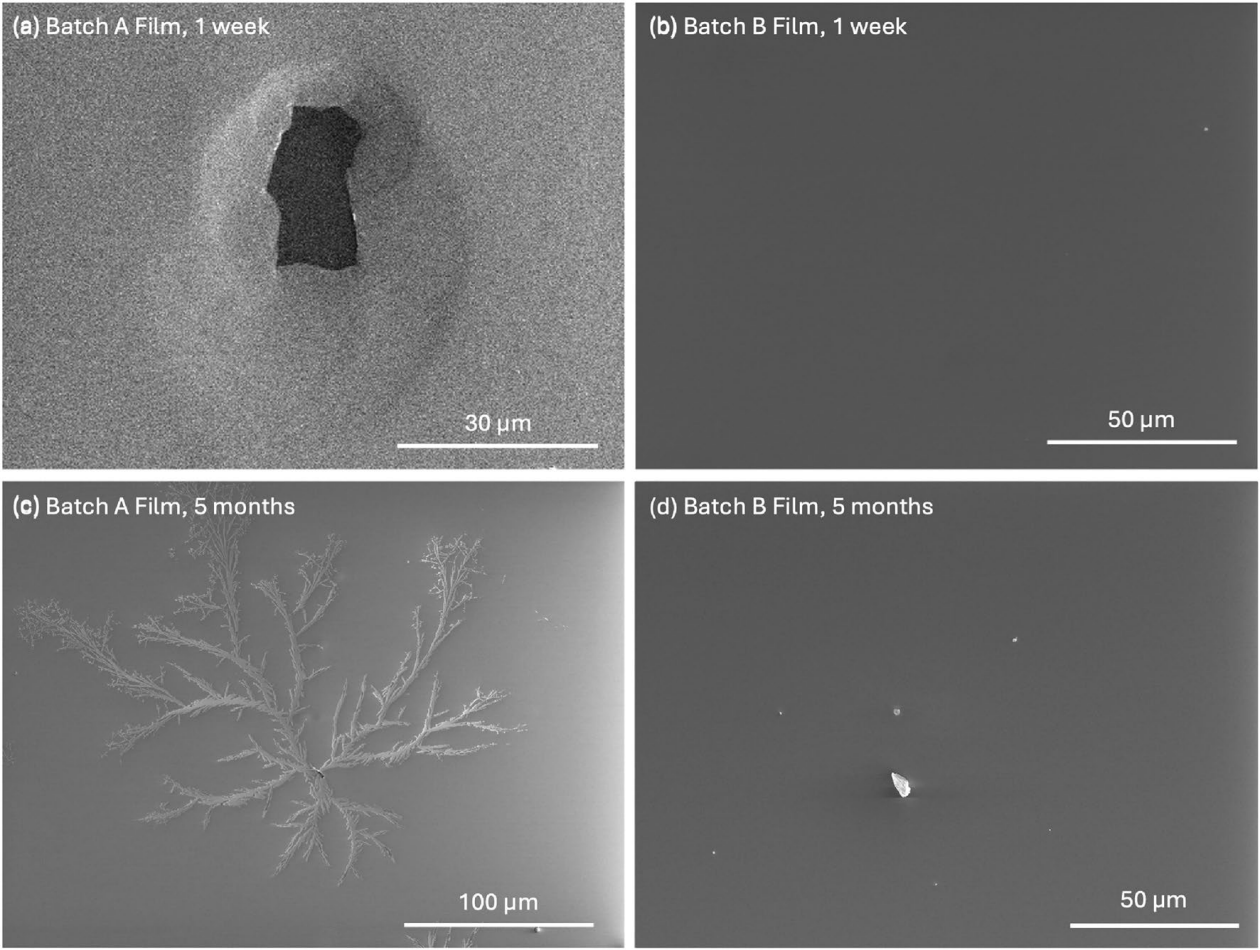
SEM of the Se–Te film surface for devices made from ** a** Batch A and** b** Batch B deposited on ITO within one week of fabrication. A small particle can be seen on the film from Batch B in the upper right, giving some reference to the surface.** c** SEM image of the Batch A film five months after fabrication, showing branching crystals across the surface.** d** Image of the Batch B film five months after fabrication, showing only particles scattered across the surface, likely from handling and storage in a non-cleanroom environment

SEM images of the surfaces of two of the fabricated films within a week of fabrication can be seen in Fig. 10a and 10b. The film made from Batch A exhibited ruptures in the film, similar to that shown in Fig. 1b, and over time developed crystal branches along the surface, as seen in Fig. 10c. After six months, the surface was fully covered in a variety of patterns. No ruptures could be found on the film deposited with Batch B, and the surface remained smooth. SEM imaging five months after fabrication revealed scuff marks and particles on the surface, such as those shown in Fig. 10d, likely due to repeated handling and testing in a variety of environments, with no crystalline patterns emerging. This compares well to aging tests performed on Se–Te by Nag and Gupta, which found no crystallization in Se–Te films over six months [36].

The structures seen in Fig. 10c provide further evidence of the formation of a crystalline Se-based material. Se has been shown to present as needle-like crystals when formed as t-Se nanoparticles or h-Se with exposure to oxides) [55, 56]. While the underside of the Se–Te film, as viewed through the transparent substrate, exhibited no observable changes, the top, air-exposed surface gradually lost its glassy quality, beginning with patches of dull, rough areas, until eventually coating the full film. This indicates macro-crystallization only at the surface, and not through the entire depth of the material. The presence of defects in Batch A, both in composition and structure, likely contributes to the formation of SeO$_2$ crystallites. Similar development of surface crystallization can be seen in unencapsulated pure and stabilized a-Se films after a period of several months, as shown in Fig. S6 of the SI, indicating that oxidation is likely accelerated from air exposure at the surface.

**Fig. 11:**
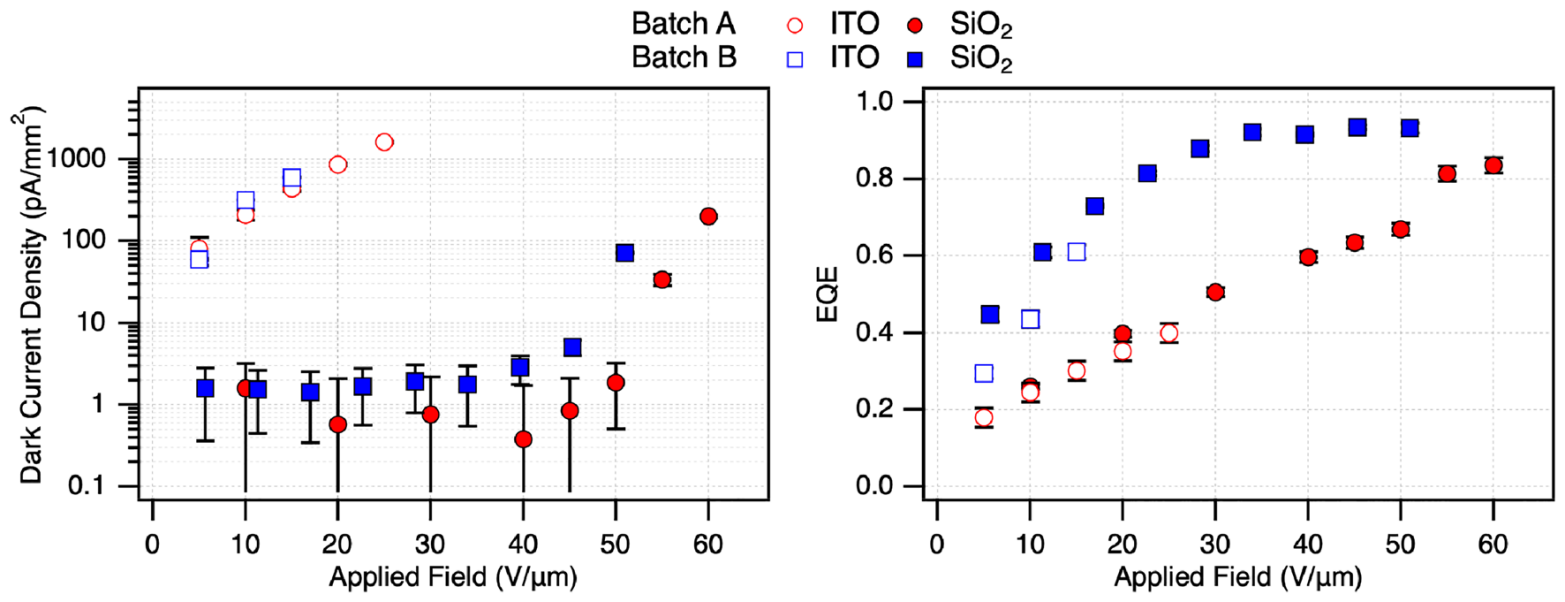
**a** Dark current and** b** external quantum efficiency of devices made from Batch A and Batch B, with and without an SiO$_2$ blocking layer. EQE was calculated from photocurrent measurements conducted with 532 nm light

The presence of oxides in such small quantities in the bulk of the films may not be expected to have a strong effect on the performance of photoconductive devices; however, a comparison of devices fabricated from Batch A and Batch B shows a significant difference in performance. Figure 11a shows the dark-current density for Batch A and Batch B devices, with and without a 30 nm SiO$_2$ hole blocking layer. Devices fabricated from Batch A exhibit slightly reduced dark current, indicative of increased effective resistivity. Oxides are typically insulating, and will be expected to increase the resistivity of the Se–Te film and suppress leakage current. In Se–Te alloys, Te incorporation typically increases conductivity and modifying the electronic structure of a-Se [13, 57]. However, oxide-related defective regions would locally disrupt this conductive network, introducing insulating phases and defect states that hinder free-carrier transport. This results in an increase in effective resistivity, despite the presence of Te, and reduces overall leakage current. Devices without a blocking layer are highly conductive, as expected for Se–Te alloys, though still less so when oxidized. The Batch B device was only able to sustain an applied field of 15 V/µm before failure, likely due to increased Joule heating associated with higher conductivity, leading to crystallization and shorting [58]. In contrast, the Batch A device sustains fields up to 25 V/µm, consistent with reduced conductivity and suppressed Joule heating under higher electric field.

The EQE for each device under green illumination is shown in Fig. 11b. Devices fabricated from Batch A contain a lower Te concentration (11.6 wt. %) than Batch B (16.3 wt. %), which would be expected to produce only a modest (3–5%) reduction in EQE at moderate fields (10–30 V/µm), insufficient to explain the substantial performance degradation observed. The Batch A devices exhibit a markedly suppressed and nearly linear EQE response with increasing field, in contrast to the exponential rise and saturation behavior characteristic of semiconductor photoconductors observed in Batch B. The Batch B device achieves peak efficiency near 35 V/µm, while Batch A devices show no saturation even at 60 V/µm.

These results suggest that oxidation introduces defect states and insulating regions that only modestly affect dark conduction but strongly impair photocarrier generation and collection. The presence of Se and Te oxides and associated oxide–amorphous interfaces likely introduces trap states that capture carriers, increase recombination, and disrupt transport pathways. While dark current can still be supported through remaining conductive Se–Te pathways and is suppressed with the presence of oxides, efficient photocurrent generation requires carrier transport across the full active volume, making it far more sensitive to localized defects. As a result, oxidation produces a relatively small reduction in dark current but a pronounced suppression in EQE. Overall, these findings demonstrate that degradation of Se–Te source materials leads to defect-mediated transport limitations that significantly impact device sensitivity and the fields required to achieve high quantum efficiency.

Bulk elemental Se and Te are generally reported to have limited reaction with oxygen and water at room temperature. SeO$_2$ is typically formed by the heating and evaporation of Se in an oxygen-rich atmosphere or oxidation through interaction with nitric acid (HNO$_3$); TeO$_2$ can similarly be formed by heating Te in an oxygen-rich environment or by dehydrating tellurous acid (H$_2$TeO$_3$), while TeO$_3$ is formed from the heating of orthotelluric acid (Te(OH)$_6$). However, a few studies focus on or discuss these issues in Se. Cornet and Rossier mention that vacuum-deposited thin films of pure selenium are “strongly catalysed by moisture,” resulting in crystallization as hexagonal-phase globules when exposed to ambient air; however, they do not elaborate on the result more than this [59]. Tanaka reviews the negative effects of oxygen impurities in pure selenium melt-quench materials on resistive properties; several studies examine the effects of doping selenium with SeO$_2$, showing that even a small addition of oxygen to pure selenium, on the order of 10 ppm, can have strong effects on transport properties and the creation of shallow trap states [60–62]. Szabo et al. studied the effect of residual gas vapors during evaporation of Se and Se–Te films, finding similar results on the changes to electrical properties of other studies for a-Se; the studies of Se–Te films did not look at the impact of oxygen or water, though they did note that the addition of Te tends to stabilize Se [63].

The apparent reactivity of Se–Te alloys may stem from their preferential generation of Se–Te–Se bonds in a chain structure, which has been shown to allow dense packing and increased microhardness in films [12, 20, 64, 65]. However, fabrication conditions play a significant role in the formation of ring or chain-like structures, especially in bulk materials [66]. While fabrication conditions for films have been optimized for structural, optical, and electronic properties, bulk preparation techniques, such as those used for pellets used in the fabrication of films, have received less attention [65, 67].

In a-Se, crystallization occurs at broken bond ends, which occur in greater numbers in linear polymeric chains as compared to ring structures [30]. Stabilized a-Se utilizes arsenic (As) and chlorine (Cl) to mitigate these broken bonds, and high-quality fabrication lends toward structures with a preference toward Se$_8$ rings. For Se–Te, increasing numbers of linear polymeric chains lead to an increase in the number of dangling bonds and nucleation points. Ward explained that with increasing concentration of Te, the strength of covalent bonding decreases, leading to shorter chains, with an increase in van der Waals forces between the chains that leads to enhanced packing [66]. Geoffrion and Guisbiers predicted through simulation that, over time, Se migrates to the surface of Se–Te compounds, disrupting molecular structures throughout the material and leaving the surface more vulnerable to external reactants and crystallization [68].

Various crystalline binary systems combining metals and transition metals with Se have been shown to develop surface oxidation under atmospheric exposure, especially over prolonged periods of storage [69–71]. The results presented in this work provide direct evidence that oxidation in Se–Te pellets is not uniform, but instead proceeds through preferential oxidation of Te at the surface, with oxygen bonding predominantly to Te and forming TeO$_x$ species, while Se remains largely in the elemental state. This behavior is consistent with prior reports of Te-containing systems, where Te is more readily oxidized due to favorable bonding energetics and surface reactivity [72, 73]. Mechanistically, oxidation is initiated at defect sites, undercoordinated Te atoms become capable of reacting with ambient O$_2$, leading to the formation of an amorphous TeO$_x$ layer at the surface. A similar defect-mediated oxidation pathway has been described in t-Te, where bond disruption generates reactive sites that enable interaction with molecular oxygen and subsequent oxide growth [74]. The absence of crystalline oxide signatures in XRD further supports that these oxides are predominantly amorphous and confined to the near-surface region, consistent with reports of amorphous TeO$_x$ formation under ambient degradation conditions [72].

As Te is preferentially oxidized and incorporated as TeO$_x$, the near-surface region becomes progressively enriched in Se. This compositional shift is reflected in the XPS-derived Se-to-Te ratios and suggests a mechanism of oxidation-driven elemental redistribution, in which Te is selectively removed from the Se–Te network. Such behavior is consistent with prior predictions of Se migration and surface enrichment in Se–Te systems over time [68]. The resulting Se-rich regions are structurally unstable in the amorphous state and can undergo local rearrangement into more energetically favorable configurations. This provides a pathway for the crystallization of a-Se to t-Se, as observed in the XRD data for both Batch A and Batch B (aged) samples, and indicates that the formation of crystalline Se is a secondary process driven by oxidation-induced segregation rather than direct oxidation of Se.

While Se oxidation is less favorable, the presence of SeO$_x$ species in Batch B (aged) sample indicates that secondary oxidation pathways do occur; the role of humidity is especially important in this process. Water has been shown to facilitate oxidation in chalcogenide systems through hydroxylation and the formation of hydrated oxide species [75]. In the case of selenium, oxidation results in SeO$_2$, which is hygroscopic and readily forms selenous acid (H$_2$SeO$_3$) in the presence of water [48]. These hydrated species are mobile and can be redistributed or removed from the surface under mildly elevated temperatures, or may dehydrate to re-form SeO$_2$. The observed decrease in the Se-to-Te ratio in the Batch B (aged) sample is consistent with moisture-assisted Se depletion, in which Se is partially oxidized and subsequently redistributed or removed via dissolution, surface diffusion, or partial volatilization under humid and mildly elevated temperature conditions. This behavior is further supported by studies showing that though Se oxidation is less favorable than Te oxidation, it can occur under conditions of increased surface reactivity and environmental exposure [73, 76, 77].

The morphological differences observed between Batch A and Batch B (aged) can be understood in terms of oxidation kinetics and structural evolution. Batch A, which was exposed to ambient conditions over several years, exhibits larger, rounded, and partially embedded surface features, suggesting prolonged cycles of oxidation, diffusion, and structural relaxation. Over these extended timescales, oxide layers can thicken and penetrate further into the surface, enabling material redistribution and the development of floret-like morphologies. In contrast, Batch B (aged), subjected to elevated humidity and temperature over a shorter period, shows smaller, sharper, and more surface-localized features. These structures are consistent with an earlier-stage oxidation regime dominated by rapid nucleation of oxide and Se-rich regions at the surface, with limited time for diffusion-driven smoothing or incorporation into the bulk. The acceleration of oxidation under humid conditions is consistent with prior observations of moisture-enhanced degradation and oxide formation in Te-containing materials [72]. While the accelerated aging process employed did not provide a direct comparison to the extended storage of the Batch A pellets, it did assist in the understanding of the mechanistic processes responsible for the Se–Te pellet evolution.

Evaporation of the defected pellets leads to disruptions in the deposited film, with metastable phases of contaminants depositing throughout, resulting in the ruptures observed either due to gas release or poor adhesion of a crystalline component to the substrate surface. The voids and compositional defects in the deposited films would generate further nucleation points, leading to continued reaction with air and the enhanced surface degradation of the films with time. Belev et al. proposed a theory in which oxygen dopants interacted with Se in thin films, which holds well for these Se–Te devices: local areas of SeO$_2$ induce trapping at the interface of the crystals and amorphous phase, and with time, SeO$_2$ forms Se=O bonds, resulting in deep traps and severely impacting transport [62]. With the inclusion of Te, this would likely evolve to the formation of TeO$_2$ instead of SeO$_2$, and even greater opportunities for the development of trap states and disturbances to transport would occur. By comparison, films deposited from uncontaminated evaporation pellets would have far fewer defects, forming the longer, well-packed polymeric chains with fewer nucleation points, as reported in other studies with extended lifetimes under ambient conditions.

This work has demonstrated that it is of great importance to employ proper storage techniques for evaporation materials, including storage in an inert atmosphere or under vacuum, and in temperature-controlled environments. Other studies have demonstrated oxide formation and crystallization of Se- and Te-containing chalcogenides under light exposure, further implying that dark storage will assist in prolonging the shelf life of the evaporation materials [74, 75]. Proper storage will result in reliable high-quality devices of Se–Te; however, further efforts should be considered to ensure the long-term viability of these devices, including encapsulation to prevent eventual oxidation of surface defects that are likely to arise over time. With proper procedures in place, detrimental oxidation can be prevented and stability may be achieved.

## Conclusion

A study of the atmospheric exposure of Se–Te evaporation materials demonstrated that oxidation of the pellet occurs over extended storage periods, resulting in the formation of amorphous TeO$_x$ and small levels of t-Se at the pellet surface. Accelerated degradation tests on new, previously unexposed material generated similar results, with differences in cluster development at the surface, likely due to the rapid growth of defects. The use of new Se–Te pellets, stored under inert gas, for device fabrication showed significant improvement in the optoelectronic response to green light and greater stability of the film surfaces when stored in ambient conditions.

The possible mechanisms for the oxidation of Se–Te were discussed, indicating a surface-driven process dominated by preferential Te oxidation, Se enrichment, and subsequent Se crystallization. This work highlights the importance of high-quality, uncontaminated evaporation materials, along with their proper storage, in the fabrication of Se–Te films and devices, particularly when developing high-sensitivity photodetectors.

## Supplementary Information

Below is the link to the electronic supplementary material.

Supplementary file 1 (pdf 15526 KB)

## Data Availability

The data presented in and that support the findings of this study will be made available upon reasonable request.
