# Supplementary material for: Exposed: investigation of oxidation in selenium–tellurium evaporation materials and its effect on optoelectronic devices
Source: J Mater Sci Mater Electron. 2026 May 28;37(15):1204. doi: 10.1007/s10854-026-17574-5 (PMC13219230; doi:10.1007/s10854-026-17574-5)
Supplement: Supplementary file 1 — (pdf 15526 KB) [file 10854_2026_17574_MOESM1_ESM.pdf]

Supplementary Information
Exposed: Investigation of oxidation in selenium-tellurium evaporation materials and its effect on optoelectronic devices

Kaitlin Hellier[1,*], Thomas D. Yuzvinsky[2], Evan Walls[3], Molly McGrath[1], and Shiva Abbaszadeh[1,*]

[1]Radiological Instrumentation Laboratory, Electrical and Computer Engineering Department, University of California, Santa Cruz
[2]Electrical and Computer Engineering Department, University of California, Santa Cruz
[3]Department of Integrated Engineering, University of San Diego
[*]khellier@ucsc.edu, sabbasza@ucsc.edu

The following figures provide additional imaging of Se-Te Batch A pellets, the full set of images for the aged Batch B pellets, and images of pure and stabilized a-Se films mentioned in the text. All are discussed in the body of the main text and are provided to support findings and comparisons.

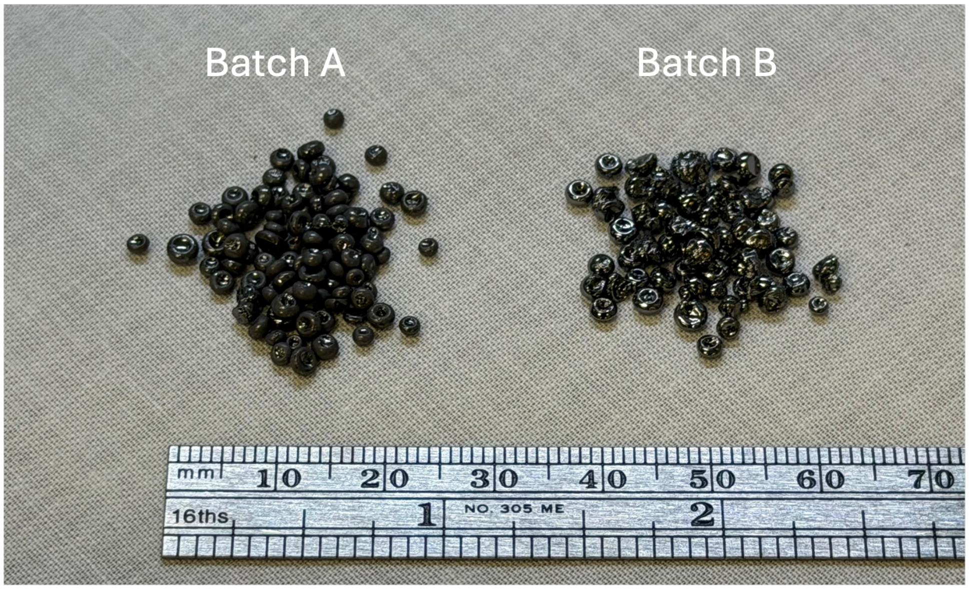

Fig. S1. Image of pre-alloyed pellets from Batch A (left) and Batch B (right), studied in their original form and utilized as evaporation materials for thin films. A ruler has been added for scale. The photo was taken with an iPhone 17 Pro with 4x magnification.

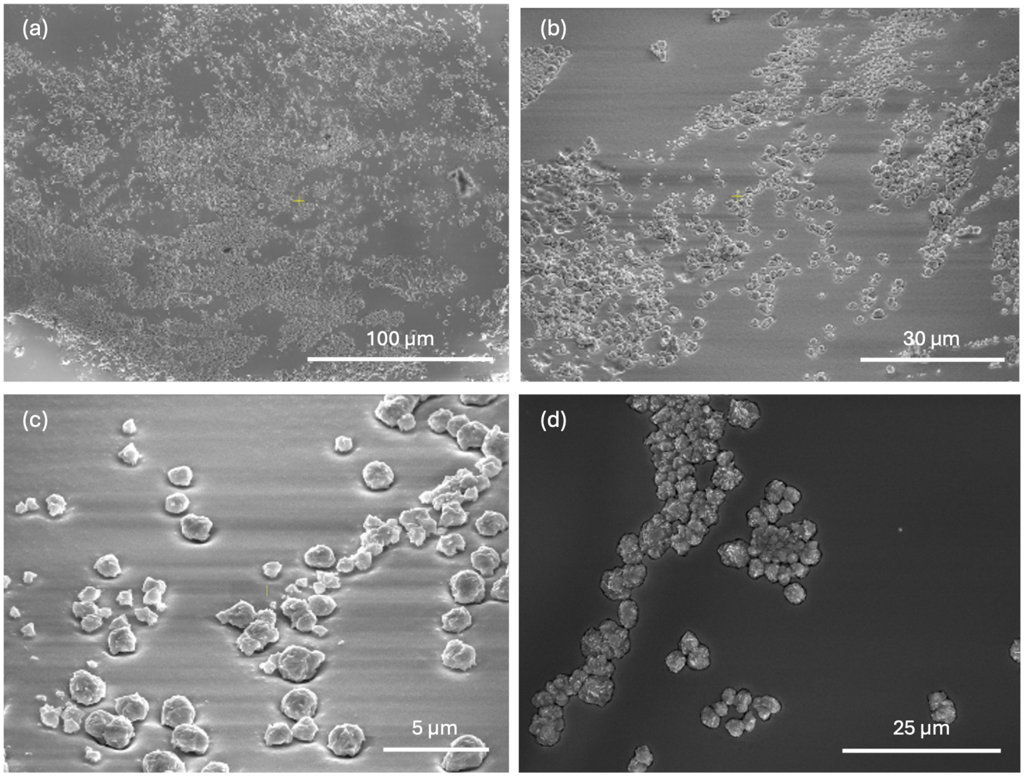

Fig. S2. Additional SEM images of Batch A pellets, demonstrating a variety of features across multiple pellets imaged.

Table S1. Weight concentrations of elements detected in each pellet by EDS, listed as a percentage of the total values. Values are averaged across a minimum of 4 measurements, which include varying points on the same sample and across multiple pellets. Batch B (aged) values correspond to measurements after 3 weeks of aging.

Sample	Se	Te	O
Batch A	75.0 ± 2.0	14.7 ± 2.0	0.4 ± 0.1
Batch A (cross-section)	69.2 ± 0.4	29.8 ± 0.4	1.1 ± 0.3
Batch B	70.2 ± 1.4	19.8 ± 1.4	-
Batch B (aged)	69.9 ± 0.8	28.2 ± 0.8	1.9 ± 0.4

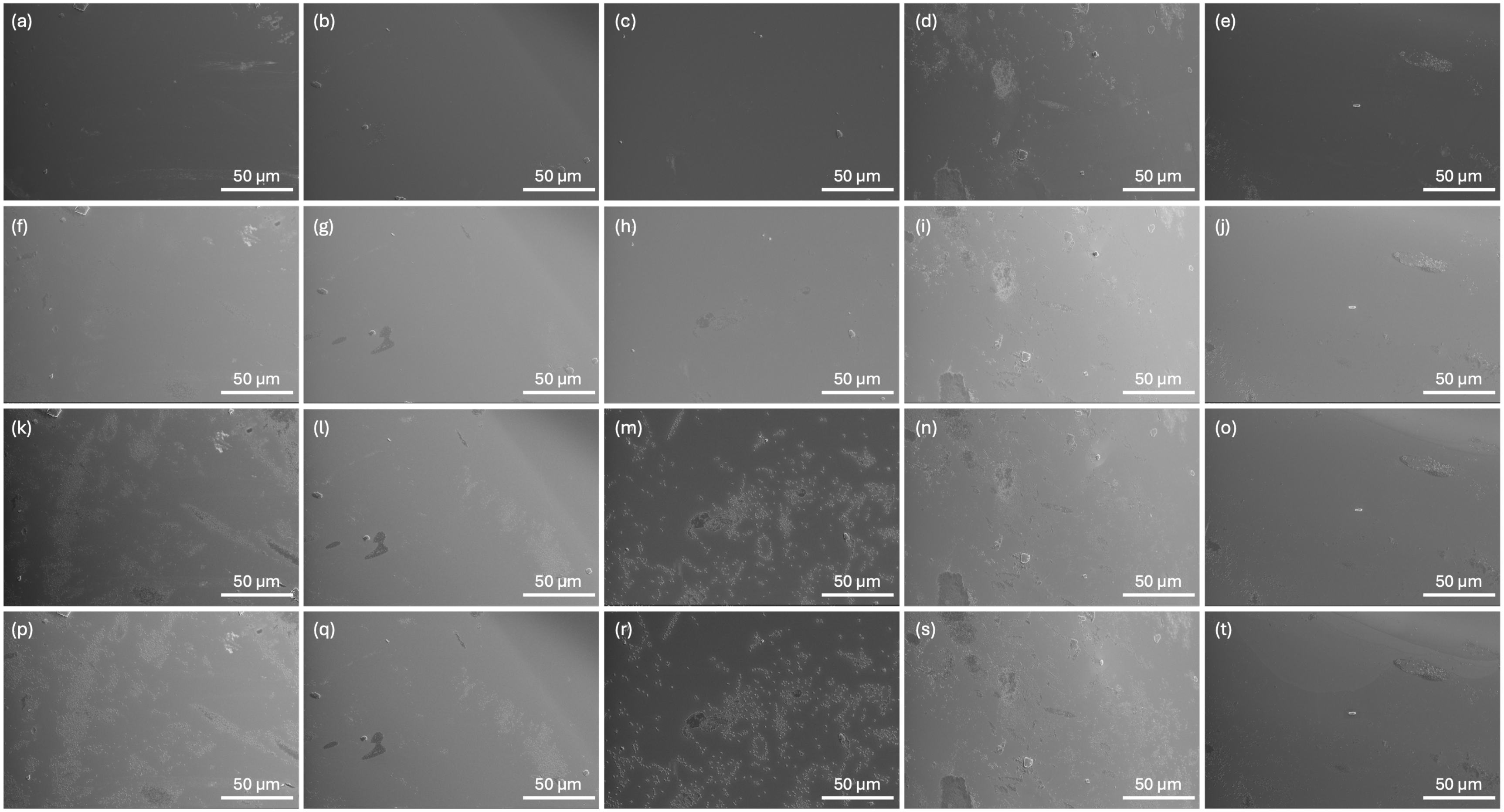

Fig. S3. Aging study of the five pellets from Batch B. (a-e) Images of each pellet before aging. Image of each pellet after (f-j) one week of aging, (k-o) after two weeks of aging, and (p-t) after one more week with no additional water added, but kept on the hotplate at 50 °C.

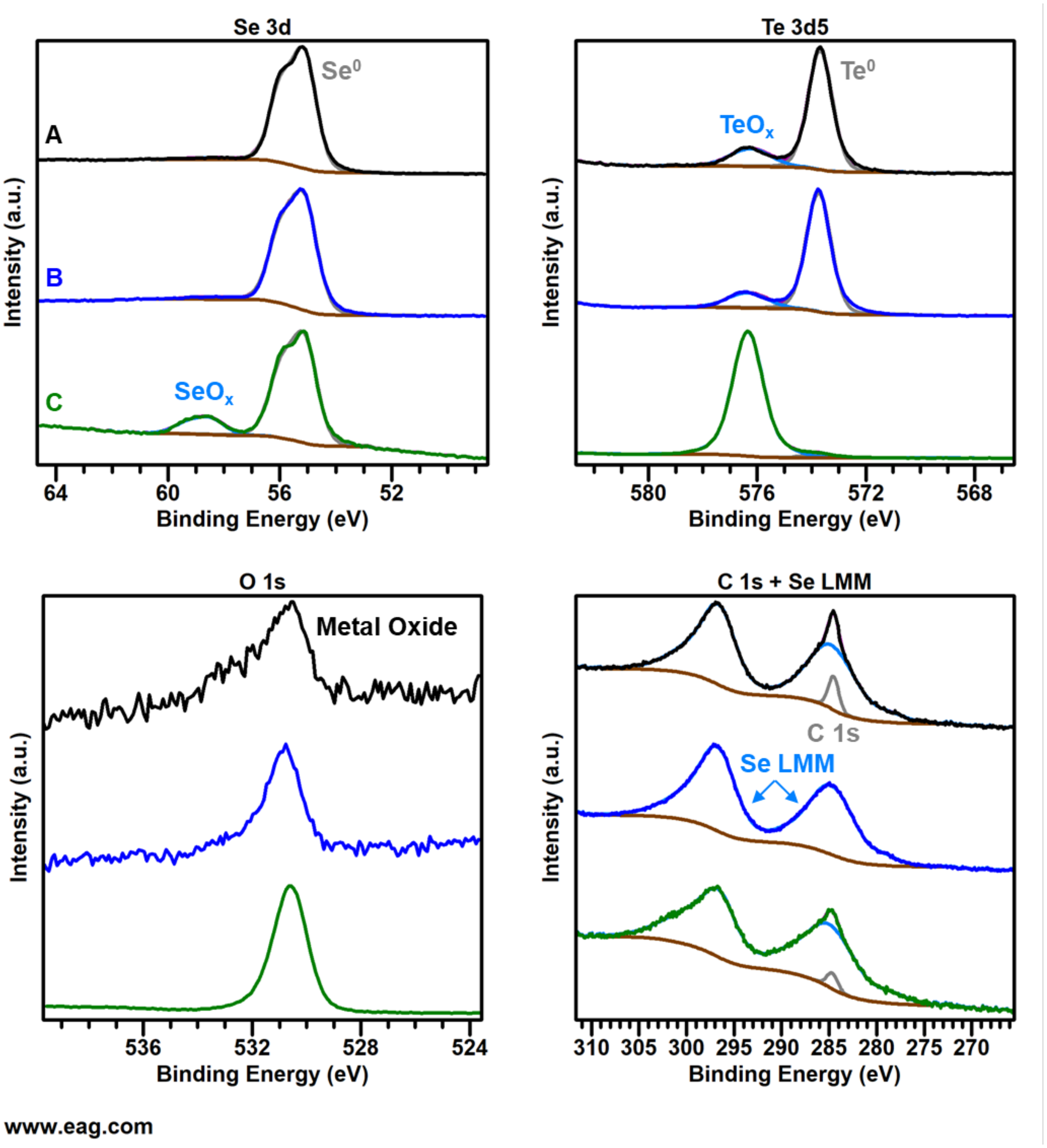

Fig. S4. Plots of the high resolution XPS scans of the three pellet batches covering the (top left) Se 3d, (top right) Te 3d5, (bottom left) O1s, and (bottom right) C1s + Se LMM binding energies. The brown lines below the scan show the background, while peak fits can be seen in light blue and grey.

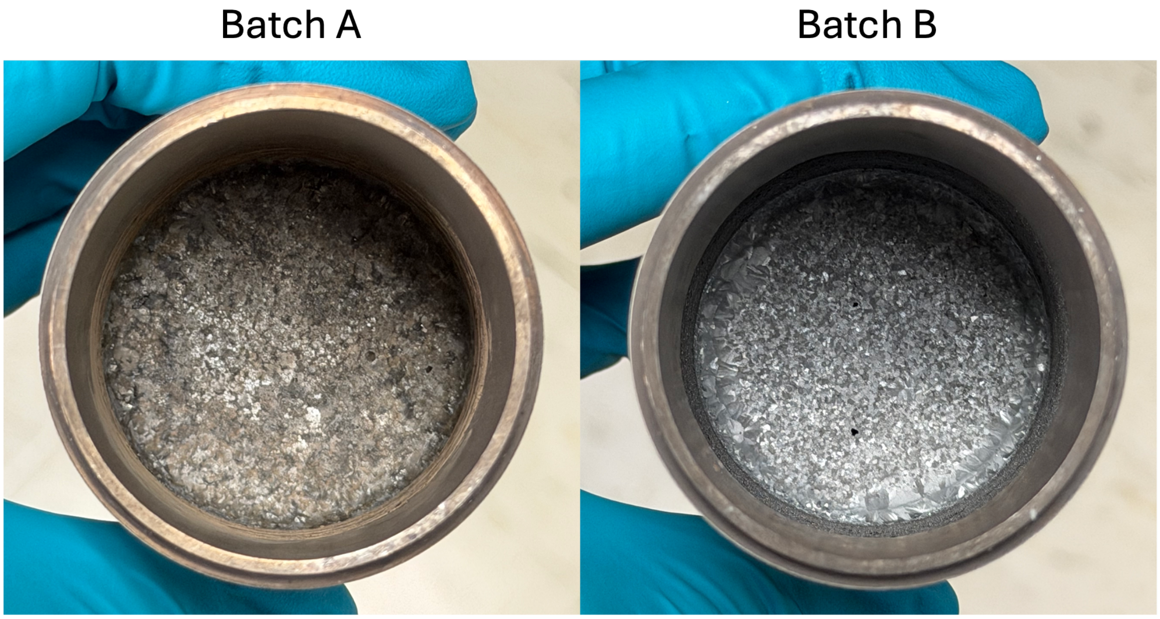

Fig. S5. Photos of the residual melt-material in the crucible for (a) Batch A and (b) Batch B pellets.

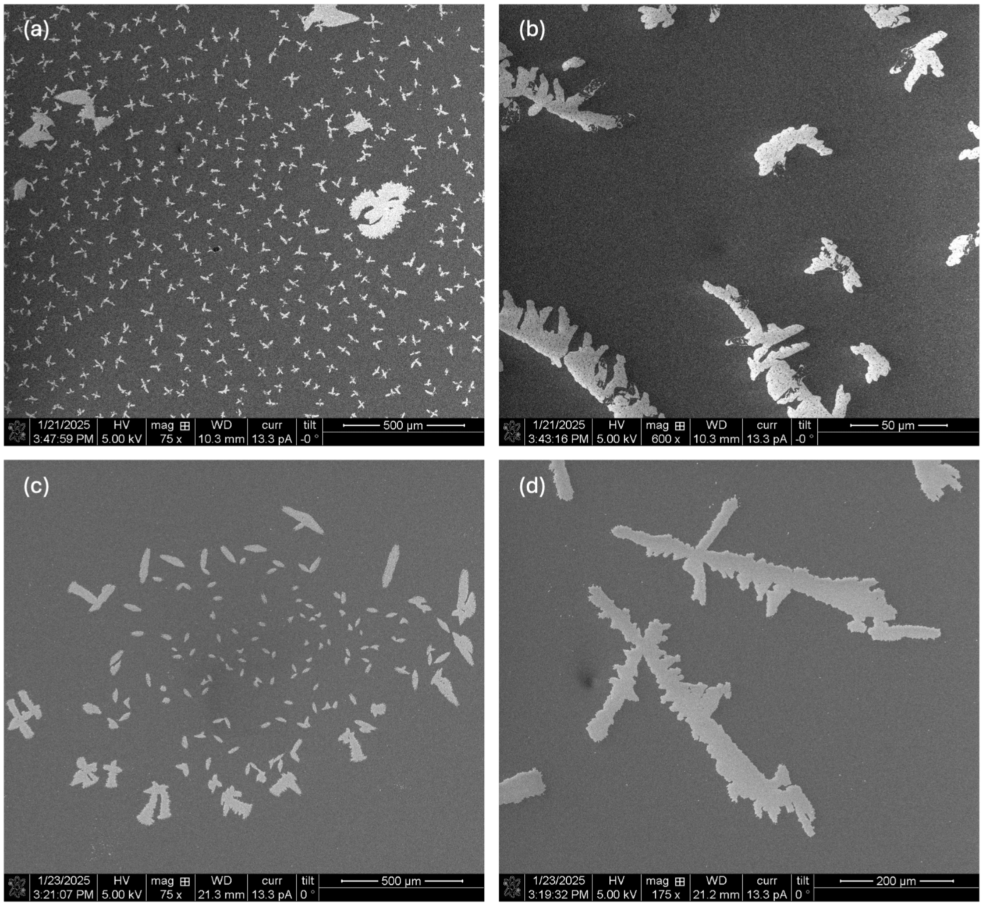

Fig. S6. SEM images of (a, b) pure Se after one month of aging and (c, d) stabilized Se after several months of aging.
